# HLA mismatches and hematopoietic cell transplantation: structural simulations assess the impact of changes in peptide binding specificity on transplant outcome

**DOI:** 10.4172/1745-7580.1000048

**Published:** 2011

**Authors:** Chen Yanover, Effie W. Petersdorf, Mari Malkki, Ted Gooley, Stephen Spellman, Andrea Velardi, Peter Bardy, Alejandro Madrigal, Jean-Denis Bignon, Philip Bradley

**Affiliations:** 1 Program in Computational Biology, Fred Hutchinson Cancer Research Center, Seattle, WA, USA; 2 Division of Clinical Research, Fred Hutchinson Cancer Research Center, Seattle, WA, USA; 3 Center for International Blood and Marrow Transplant Research, USA; 4 European Group for Blood and Marrow Transplantation, Italy; 5 Australian Bone Marrow Donor Registry, Australia; 6 Anthony Nolan, UK; 7 Blood Bank Nantes and SFGM, France

## Abstract

The success of hematopoietic cell transplantation from an unrelated donor depends in part on the degree of Human Histocompatibility Leukocyte Antigen (HLA) matching between donor and patient. We present a structure-based analysis of HLA mismatching, focusing on individual amino acid mismatches and their effect on peptide binding specificity. Using molecular modeling simulations of HLA-peptide interactions, we find evidence that amino acid mismatches predicted to perturb peptide binding specificity are associated with higher risk of mortality in a large and diverse dataset of patient-donor pairs assembled by the International Histocompatibility Working Group in Hematopoietic Cell Transplantation consortium. This analysis may represent a first step toward sequence-based prediction of relative risk for HLA allele mismatches.

## Background

Unrelated hematopoietic cell transplantations (HCTs) involving perfectly matched patient-donor pairs generally have higher success rates than those between patients and donors mismatched at one or more loci [1-4]. As HLA-matched donors are only available for a minority of patients, there is considerable interest in distinguishing HLA mismatches that significantly increase the risk of complication (nonpermissive mismatches) from those that do not (permissive mismatches). One approach has been to look at HLA mismatches in terms of the set of amino acid mismatches present, with the goal of identifying specific amino acid mismatches that are associated with increased risk [5,6]. Knowledge of general patterns at the level of amino acid mismatches could allow estimation of risk even for allele-level mismatches with insufficient prior clinical data. Here we investigate the connection between HLA mismatching and transplant outcome, using structural predictions of HLA-peptide interactions to characterize the impact of amino acid mismatches on peptide binding specificity divergence, a potential mediator of T-cell alloreactivity. Using a large and diverse clinical dataset assembled by the International Histocompatibility Working Group in Hematopoietic Cell Transplantation consortium, we find support for the hypothesis that residue-level mismatches predicted by structural modeling to perturb HLA-peptide binding specificity are associated with increased mortality risk.

To predict the effect of a single amino acid mismatch on HLA-peptide binding specificity, we build structural models for a pair of HLA sequences that differ only by the mismatch in question. One sequence is taken from a naturally occurring HLA allele; the other is a mutated variant which may not match the sequence of a known allele. Flexible-backbone peptide docking simulations are performed against each of these protein models, and binding specificity profiles are inferred from the output of these simulations. The magnitude of the difference between these predicted binding profiles is taken as the predicted specificity divergence induced by the given mismatch.

To assess the impact of binding specificity changes on transplant outcome, we compared predictions of induced binding specificity divergence for a set of common amino acid mismatches in the HLA-C locus with mortality risk factors for those mismatches estimated from the clinical dataset. This comparison identified a significant correlation between the magnitude of the binding specificity change induced by a mismatch and the mortality risk associated to that mismatch. The validity of this analysis depends in part on reasonably accurate estimates of peptide binding specificity for HLA sequences and their mutated variants. To test the robustness of the observed correlation, we recalculated the binding specificity divergences using the neural network peptide binding predictor NetMHCpan-2.4 [7], and found again a statistically significant correlation with mortality risk. Our choice of the *pan-specific* predictor NetMHCpan — which is capable of making predictions for novel HLA sequences by generalizing from experimental binding data for related alleles — was motivated by the need to make predictions for HLA-C alleles and allele-variants with little or no experimental binding data. The choice of a structural approach to HLA mismatching was also motivated in part by this lack of experimental binding data: our structure-based binding predictions rely on estimates of peptide-HLA binding affinities derived from physico-chemical potential energy functions and atomically-detailed molecular models. Furthermore, atomic level simulations of HLA-peptide complexes provide additional valuable information beyond binding affinity [8], such as structural insight into differences in binding specificity and changes in peptide binding modes, also thought to be a factor in T-cell alloreactivity [9].

Structural features have been used previously to quantify mismatching between HLA molecules [10-12]. Although structure-based algorithms have been applied successfully in certain clinical settings [13], studies have questioned their effectiveness in the context of HCT [14,15]. Kawase and co-workers identified six amino acid mismatches associated with increased risk of acute graft-versus-host disease [6]. They then characterized these mismatches with respect to three physicochemical properties of amino acids: hydropathy, isoelectric point, and molecular weight, and found that four of the mismatches were characterized by a large change in hydropathy, while a fifth resulted in a significant change in the molecular weight of the mismatched amino acid. The predictive value of these physicochemical properties in distinguishing permissive from non-permissive amino acid mismatches was not assessed, however.

## Results

As described in detail in the methods section, we assigned a relative mortality risk factor to each residue-level mismatch in the HLA-C locus based on analysis of 1,110 unrelated patient-donor HCT pairs. For example, we assigned a risk factor of 1.21 to the mismatch F versus Y at position 99, and a risk factor of 0.92 to the mismatch of G versus R at position 91. These relative risk factors compare the mortality risk for patients with the given mismatch to the mortality risk for patients without that mismatch but still mismatched elsewhere at the HLA-C locus. All patient-donor pairs analyzed were perfectly matched at HLA-A, B, DRB1, DQB1, and the other HLA-C allele (termed 9/10 matched pairs). To reduce noise in these averaged risk factors, we restricted our analysis to mismatches with high counts in the clinical dataset (details in Methods).

We then used structural modeling methods to analyze the impact of each mismatch on peptide binding. We computed peptide-binding specificity profiles for an HLA-C protein containing one of the two mismatched residues at the relevant position, and for that same protein after mutating that position to the other residue. We compared the resulting profiles to yield a prediction of binding specificity divergence associated with that mismatch. This structural modeling calculation was done using as a template protein both of the two HLA-C proteins with solved X-ray crystal structures, C*03:04 and C*04:01 (PDB ids 1efx [16] and 1qqd [17], respectively).

**Figure 1:**
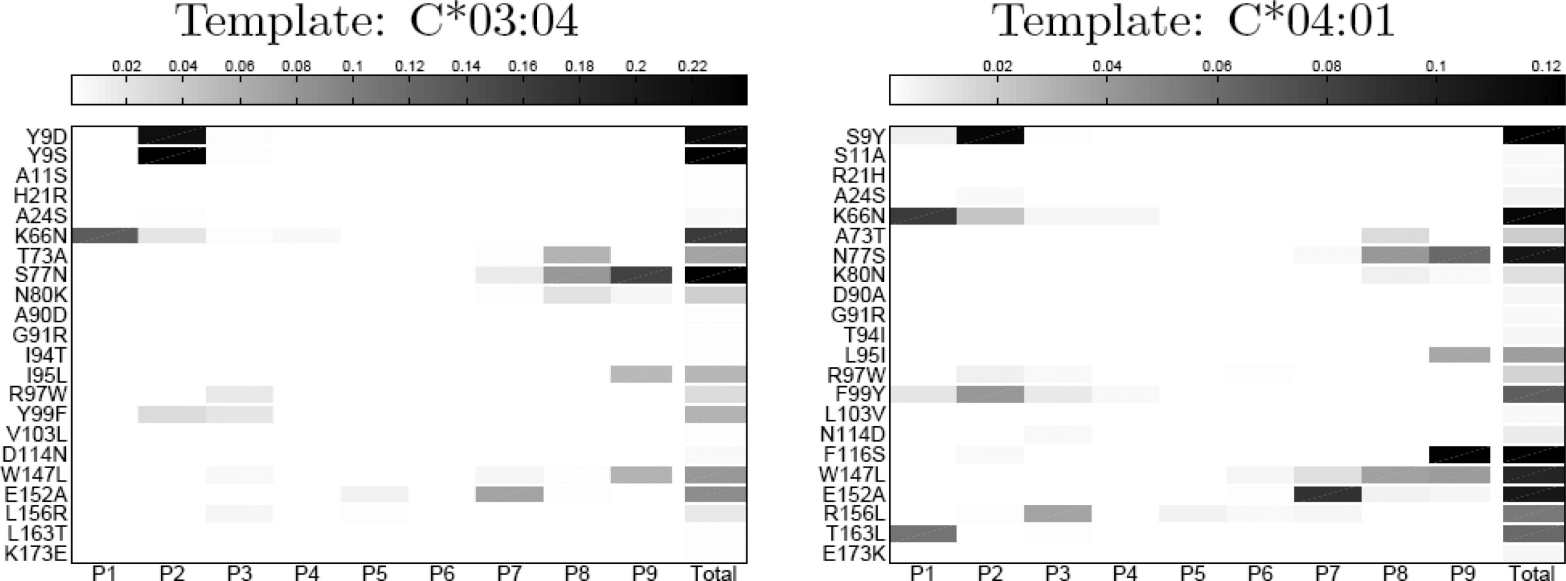
Structure-based predictions of peptide binding specificity divergence. For each analyzed mismatch, the per-position binding profile divergence induced by that mismatch is indicated by the shade of gray in the corresponding box (white indicates no change; darker colors reflect positions at which the amino acid preferences are significantly changed by the mismatch), and the predicted total change in binding specificity is shown in the final column.

**Figure 2:**
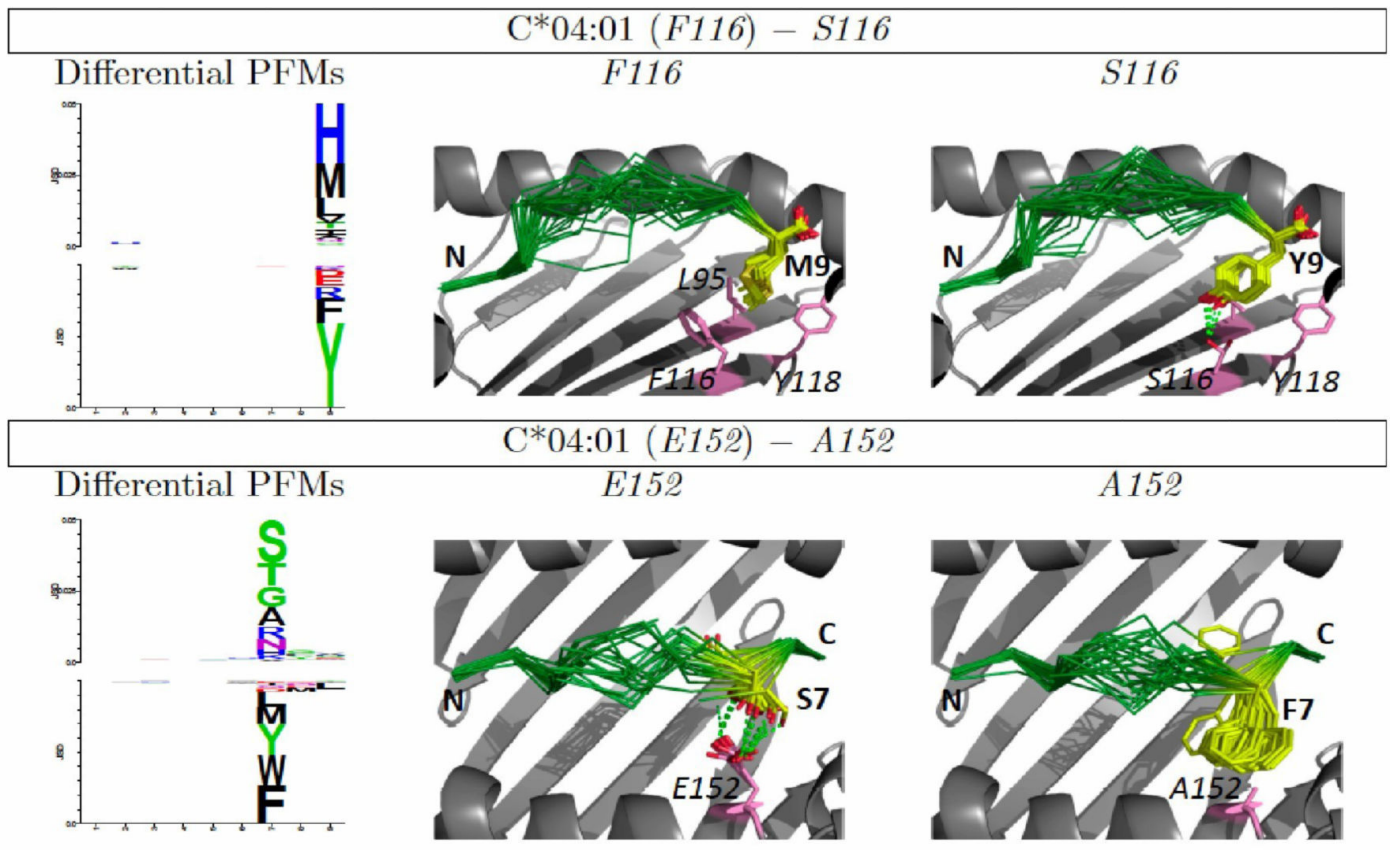
Structural basis of binding specificity divergence. Left: Differential PFMs [8], showing, for each position, the amino acids predicted to bind one HLA variant more favorably than another. The height of each column is proportional to the specificity divergence at that position; the difference in amino acid frequencies determine the height and order of the corresponding letters. Mutation of position 116 from F to S reduces preference for H, M, and L at peptide position 9 and increases preference for Y and F (upper panel); mutation of position 152 from E to A reduces preference for small polar amino acids at peptide position 7 while increasing preference for aromatics (lower panel). Structural models (second and third columns) suggest plausible connections between HLA sequence mismatches and peptide binding preference. **Y9** can form hydrogen bonds with *S116*, and **S7** with *E152*; hydrophobic interactions drive preferences of proteins with *F116* and *A152* (peptide backbone is shown in green, interacting peptide and HLA side chains are depicted in yellow and pink, respectively; for clarity, peptide positions are denoted in bold while HLA positions are italicized).

Figure 1 summarizes the results of these binding specificity calculations for the two templates. For each residue mismatch, the impact of that mismatch on amino acid preferences is shown for each of the nine peptide positions. The total predicted change in specificity — the sum of the nine positional changes — is shown in the final column. From these plots one can see that the specificity changes tend to be localized to a single position or a few consecutive positions. Some mismatches, such as 9 S/Y, affect the N-terminus of the peptide, while others, such as 77 S/N, affect the C-terminus, and these patterns generally reflect the location of the corresponding HLA positions relative to the peptide. For example HLA position 9 forms part of the pocket for the first anchor position (P2), while position 77 makes contacts with the C-terminus of the peptide. These specificity changes can be further investigated by examining the structural models produced by the binding prediction algorithm. Figure 2 illustrates structural interactions underlying the predicted changes in peptide binding specificity for two mismatches with large predicted divergences, 116 F/S and 152 E/A.

**Figure 3:**
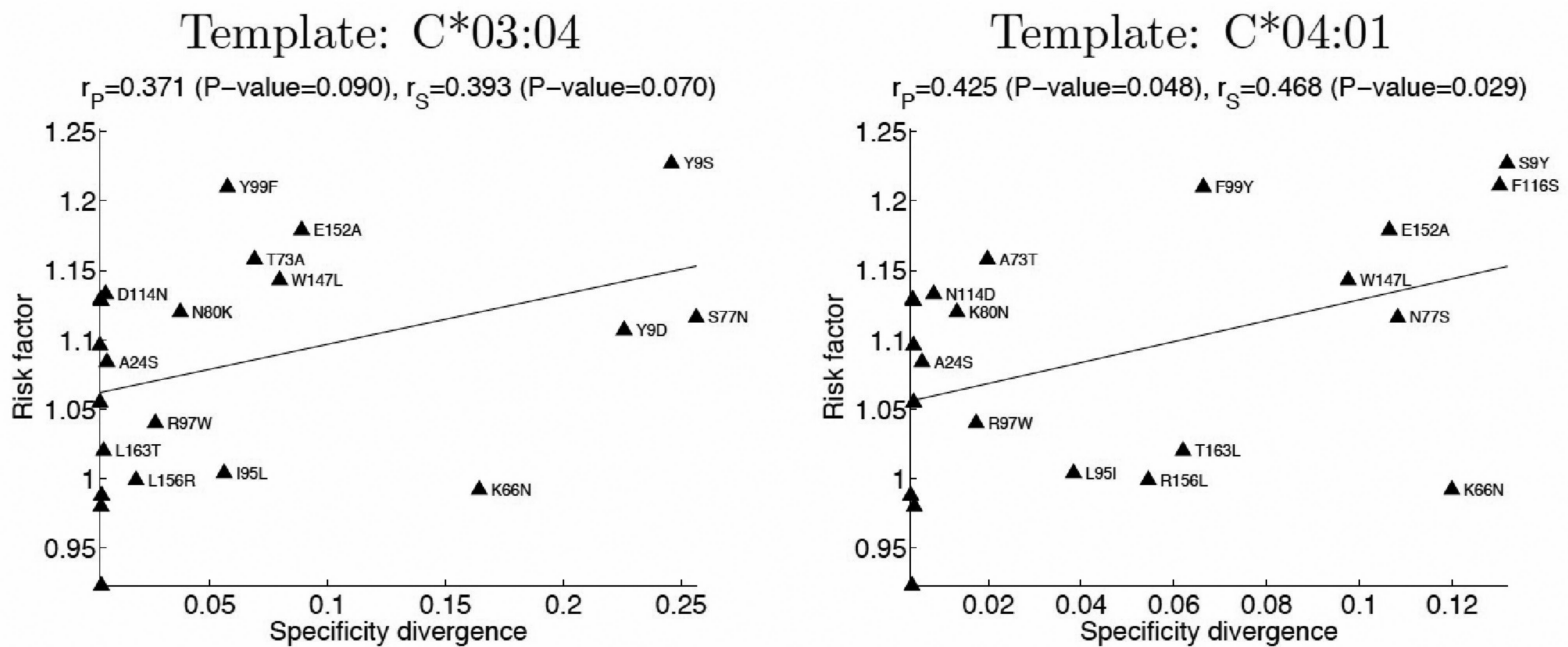
Correlation between clinical risk factor and structure-based predictions of peptide binding specificity divergence for single amino acid mismatches (Pearson's linear correlation coefficients (*r_P_*), Spearman's rank correlation coefficients (*r_S_*), and the corresponding P-values are indicated).

We next asked whether amino acid mismatches predicted to induce larger changes in binding specificity were associated with increased clinical risk factors. Figure 3 shows a plot of the relative mortality risk for each mismatch (y-axis) versus our structure-based prediction of peptide binding specificity divergence (x-axis; Supplementary Table S1). Although far from perfect, there is a clear correlation between the two measures, with Pearson's linear correlation coefficients of 0.371 (P-value 0.09) and 0.425 (0.048) for the predictions made using C*03:04 and C*04:01 structures, respectively, as template. This suggests that divergence in peptide-binding specificity may be a contributor to mortality risk after transplantation, perhaps through alloreactivity of donor T-cells towards peptides presented by patient HLA molecules but not by donor antigen presenting cells during T-cell development in the thymus. We find this degree of correlation promising for several reasons. First of all, the structural modeling, while state-of-the-art, has significant limitations (see Discussion) that may impact the accuracy of our binding predictions. In addition, the peptide-binding specificity change induced in a single template, even if correctly predicted, will not necessarily correlate with binding specificity changes induced in other templates with different intrinsic binding preferences, whereas the clinical risk factors represent averages over many different allele-level contexts for each mismatch. At the same time, none of the residue-level mismatches occur in isolation in any given patient-donor pair; they co-occur with other amino acid mismatches that may also affect outcome. Some of these correlations can be quite strong: the 77 N/S and 80 K/N mismatches co-occur across almost the dataset, as do the 103 L/V and 173 E/K mismatches. This complicates the attribution of risk to individual mismatches. We predict a larger effect for mismatches at position 77 than for mismatches at position 80, whereas their clinical risk factors are essentially identical (thus even a perfect prediction of mismatch-induced specificity divergence would correlate poorly with risk when restricted to these positions). Lastly, the extent to which per-position specificity divergences (Figure 1) affect the mortality risk is, likely, position-dependent, quantitatively similar changes in binding preference at two peptide positions might be related to different, if not opposing, clinical outcomes.

## Disease severity

**Figure 4:**
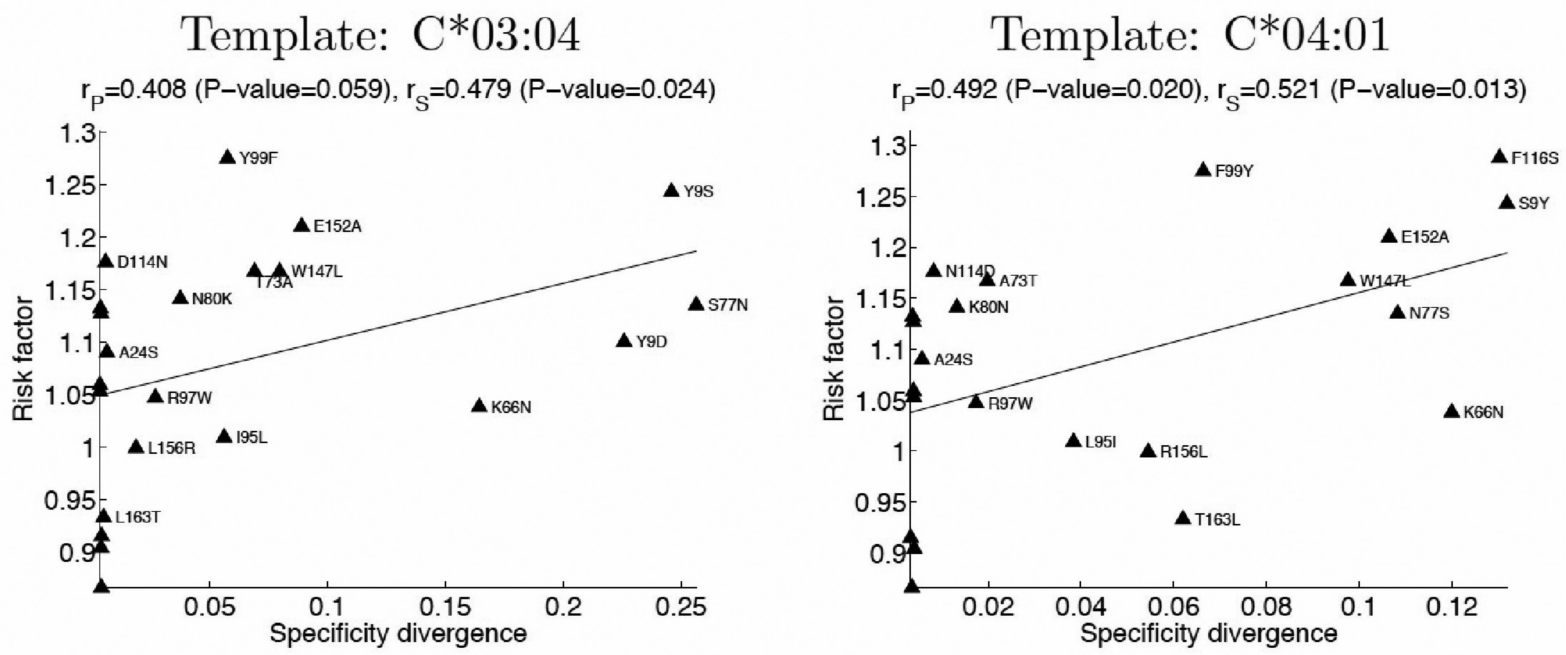
Refined clinical risk factors (excluding high-risk patients) show improved correlation with structure-based predictions of binding specificity divergence.

In the preceding analysis, the risk factor associated with a given mismatch is simply the ratio of the mortality rate for patients with that mismatch to the mortality rate for patients without the mismatch. To minimize the impact of non-genetic factors, we have focused on frequently occurring mismatches; mortality rates assigned to these mismatches are calculated over large sets of patients and are therefore less sensitive to stochastic noise. A more refined statistical assessment of the mortality risk associated with residue-level mismatches would take into account disease severity at the time of transplantation, correlations between HLA sequence positions, treatment regimen, and other relevant clinical factors. Although such an analysis is beyond the scope of this article — the primary goal of which is to present our structural approach to HLA mismatching — we sought to assess the potential effect of a more refined analysis on the observed correlations. Given that disease severity is an important non-genetic factor influencing patient mortality, we recalculated risk factors for our set of high-counts mismatches after removing all patients with severe disease (based on a three-level classification of disease severity). Encouragingly, we found improved correlations between these revised mortality risk factors and the specificity divergences predicted from simulations (Figure 4). While not conclusive, this result points to the benefits of future statistical analysis residue-level mismatching, and further supports a role for binding specificity divergence in transplant outcome.

## Comparison with NetMHCpan

**Figure 5:**
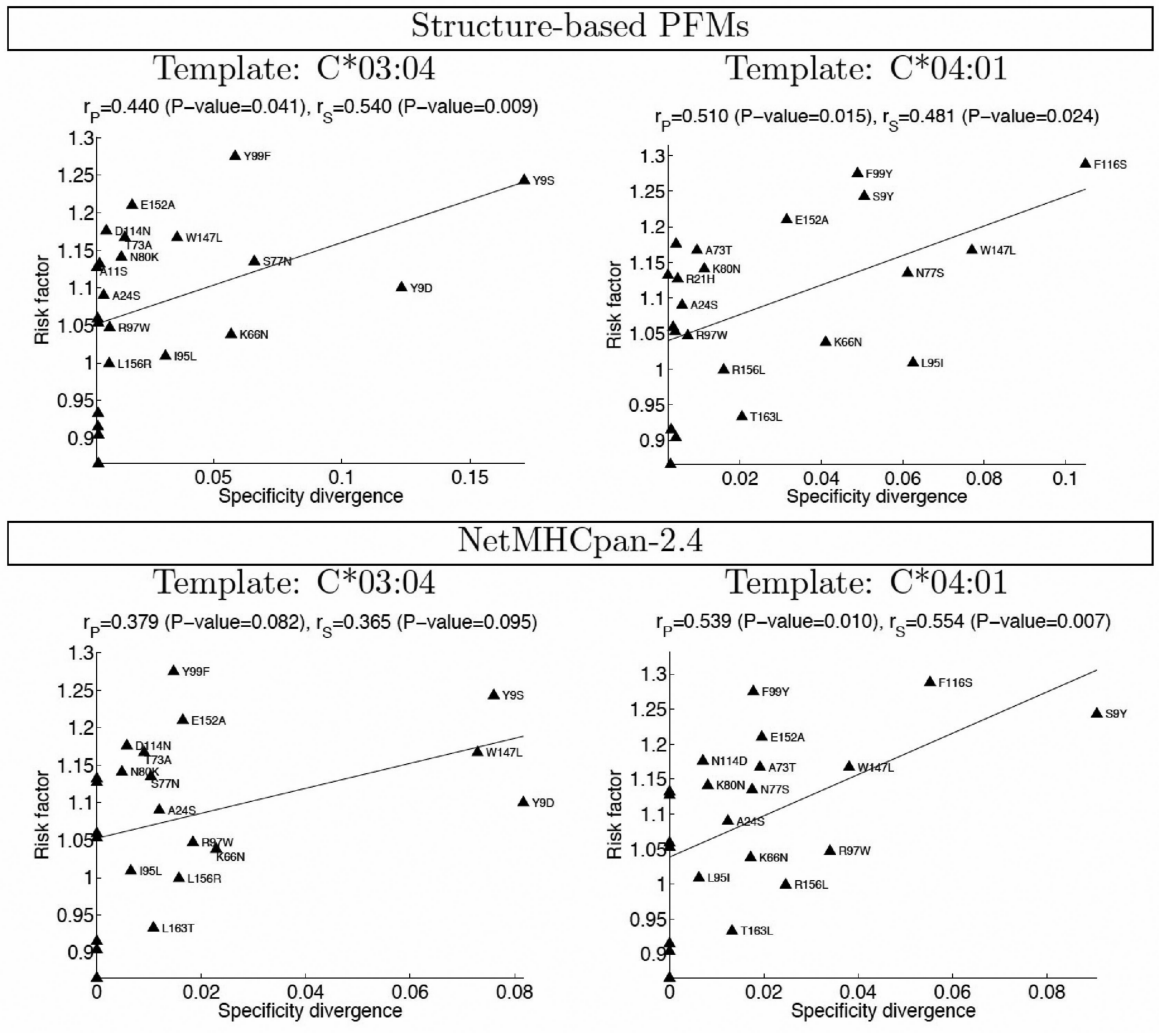
The correlation between NetMHCpan-2.4 predicted divergence and refined clinical risk factor (lower panel) is similar to that obtained using structure-based PFMs (upper panel). Divergence is defined, for both methods, in terms of the Pearson's correlation coefficient between predicted affinities for the wild type protein (either C*03:04, left, or C*04:01, right) and a protein mismatched at a single position.

The divergence in peptide binding specificity between two HLA molecules can be also inferred from predictions of peptide binding affinities for the two proteins. Unlike binding profile divergence, such a measure does not assume an underlying binding model; thus it allows a direct comparison between different prediction methods. Correlation of predicted peptide binding affinities was used by Nielsen *et al.* [18] as a distance measure to cluster HLA proteins according to their specificity profiles. Here, it is used to compare the structure-based predictions with those obtained using NetMHCpan-2.4, a state-of-the-art, neural-network-based predictor [7]. Specifically, we predicted binding affinities for a set of 10,000 random peptides to the native C*03:04 or C*04:01 sequence as well as to sequences mismatched at a single position, using either NetMHCpan or the structure-based PFMs. Following Nielsen *et al.* [18], we then defined the divergence associated with a mismatch as $1-r_{P}^{Affinity}$, where $r_{P}^{Affinity}$ denotes the Pearson correlation between the sets of predicted binding affinities for the “wild type” and the mismatched sequences. The structure- and NetMHCpan-based divergences are highly correlated (Pearson correlation coefficients of 0.802 and 0.611 for C*03:04 and C*04:01, correspondingly), attesting to the validity of the structural simulations. Importantly, the correlations between predicted divergence and mortality risk for these two approaches are similar (Figure 5), supporting the importance of peptide binding specificity divergence in transplant outcome. It should be emphasized that the NetMHCpan software was developed for the purpose of predicting binding of peptides to naturally occurring HLA alleles as opposed to the singly-mutated protein sequences necessarily used here in order to estimate the impact of individual residue mismatches. An analysis of binding specificity divergence focused on allele-level rather than amino-acid mismatching — while not directly comparable to the structural simulations — might be expected to take greater advantage of this widely used software package. Such an analysis is complicated, however, by the challenge of assigning reliable clinical risk factors at the allele level, rather than the residue level, since the patient-donor counts are much lower (none of the allele-level pairs meet our high-counts threshold of 100 mismatches in each orientation; only three allele-pairs exceed 20 mismatches).

## Discussion

In this study, we have used structural simulations to investigate the role of peptide binding specificity divergence in transplant outcome. By analyzing a large set of patient-donor pairs, we assigned mortality risk factors to individual amino acid mismatches in the HLA-C gene. We then used structural modeling simulations to predict the binding specificity changes induced by each of these mismatches. We found significant correlations between the magnitude of these predicted specificity changes and the mortality risk factors derived from the clinical data-set. For comparison, we also computed binding specificity divergences for all mismatches using the neural-network predictor NetMHCpan-2.4. These predicted specificity divergences also correlate with mortality risk, further supporting the role of peptide binding divergence in transplant outcome.

Structural modeling offers several advantages as a tool for investigating HLA-peptide interactions. The predicted divergences in binding specifity can be decomposed into positional contributions (Figure 1), and the structural basis for these positional contributions can be explored by analysis of structural models of low-energy HLA-peptide complexes with and without the mismatch of interest (Figure 2). Extending the work presented here, the impact of mismatches on HLA-KIR and HLA-TCR interactions could be examined using existing structural data for these complexes. These peptide-HLA binding simulations also have important limitations: the HLA backbone is held fixed throughout the simulations (the sidechains near the peptide can rearrange); the force fields that are used were parameterized for monomeric protein structure prediction and design, and likely could be substantially improved for modeling protein-peptide interactions; modeling simulations focused on binding of 9-mer peptides, and — in order to focus conformational sampling — the peptides were initially constrained to sample canonical positions for the anchor residues (positions 2 and 9, although these could shift during the high-resolution refinement stage), potentially skewing the results if peptides with non-canonical binding are important. We are currently working to extend the HLA-peptide modeling simulations — by incorporating limited HLA backbone flexibility, for example — to address these limitations and thereby more accurately model changes in HLA-peptide binding preferences.

Critical for clinical application would be a method for arriving at predictions of risk for allele-level mismatches. If sufficiently robust and accurate, this might allow ranking of candidate donors in cases where a perfectly matched donor cannot be found. The simplest approach would be to add the predicted binding specificity divergences for each amino acid mismatch found in the allele-level mismatch of interest to arrive at a single prediction of total binding divergence. Examination of the patterns of binding specificity divergence per peptide position shown in Figure 1 suggests one subtlety that should be considered in combining predictions: mismatches that affect the same peptide positions would be expected to combine differently from those that affect different positions. For example, if two mismatches both perturb binding at position 2, then the combined effect will likely be less than the effect of combining a mismatch that perturbs binding at position 2 with one that perturbs position 9. In the latter case, both positions 2 and 9 will likely be perturbed (hence summing the predictions might be appropriate), whereas in the former case the combined effect might be closer to taking the maximum of the two individual predictions.

In addition to predicting risk factors for patient-donor allele mismatches, we anticipate that further molecular modeling of HLA-peptide interactions, together with more sophisticated analysis of clinical risk factors in the dataset, may lead to insights into the mechanisms of graft -versus-host disease, graft rejection, and related complications. By analyzing peptide binding specificity changes independently from changes to the HLA-TCR and HLAKIR interaction surfaces, structural analysis has the potential to probe the molecular basis of clinical outcomes, generating experimentally testable hypotheses regarding disease processes.

## Methods

In order to investigate the effect of divergences in HLA-peptide binding specificity on transplant outcome, we applied a structure-based protocol for predicting peptide-HLA interactions to a large set of variant HLA molecules derived from HLA-mismatched patient-donor pairs. We sought to test the hypothesis that patient-donor pairs whose mismatched alleles are predicted to have divergent peptide-HLA binding specificity might be at greater risk for negative outcomes after transplantation. To compare structural modeling to clinical data, it was necessary to reduce the clinical data and patient-donor genotypes data down to a manageable set of relatively robust statistics. To do this, we reduced each patient/donor pair to a set of mismatched residues together with a binary survival outcome. For each residue mismatch (for example, A/T at position 73) we combined outcomes data from all patient/ donor pairs having that mismatch in order to arrive at a single statistic for the relative risk associated with that mismatch (details and rationale are given below). To make a structure-based prediction of the degree of change in peptide binding specificity associated with that mismatch, we performed peptide binding simulations on proteins having either of the two mismatched residues at the position of interest (for example, either an A or a T at position 73), and on those same proteins after introducing the mutation of interest in the HLA sequence. We compared the peptide binding specificity profiles before and after performing the mutation using a standard distance metric for probability distributions; the numerical result of this comparison was taken as the structural prediction of the magnitude of peptide binding specificity change associated with that mismatch.

## Structure-based prediction of peptide binding profiles

To generate a peptide binding specificity profile for a given HLA molecule, we perform twenty thousand independent, flexible-backbone peptide docking simulations. These simulations differ from standard docking simulations — which seek to predict the bound structure of a complex from the structures of the individual components [20] — in that the sequence of the peptide, as well as its internal structure and orientation relative to the HLA molecule, is optimized during the simulation. This sequence optimization is accomplished by incorporating protein design algorithms [21] into the docking protocol. By combining simultaneous sequence and structure optimization we can identify high-affinity peptides with a variety of binding modes. The final, optimized peptide sequences are compiled into a position-specific frequency matrix (PFM) that serves as our representation of the predicted binding specificity.

Each independent modeling simulation proceeds in two stages. In the first stage, a low-resolution backbone model for the peptide bound to the HLA is built by a Monte Carlo simulation that combines techniques from protein-protein docking, loop modeling, and *de novo* structure prediction. The peptide sequence is randomized at the start of each simulation and held fixed throughout the low-resolution simulation. The backbone of the peptide is built outward from the two canonical anchor positions (residues 2 and L for a peptide of length L) by assembling 3-residue fragments from proteins of known structure and similar local sequence (fragment assembly [22]); a break in the peptide backbone is introduced at a randomly selected location between the two anchor positions to allow independent sampling of the two halves, and loop closure algorithms are applied to close the chainbreak after each sampling step [23]. The orientation of the anchor positions is sampled by docking moves that replace the orientation in the current model with an orientation derived from a peptide-HLA complex of known structure. This guarantees that the anchor residues will sample canonical pockets in the HLA. The low-resolution simulation is conducted with a backbone-only representation of the peptide and HLA, in the context of a knowledge-based scoring function that incorporates residue environment and pair interaction preferences and a soft van der Waals term to prevent atomic overlaps [22].

**Figure 6:**
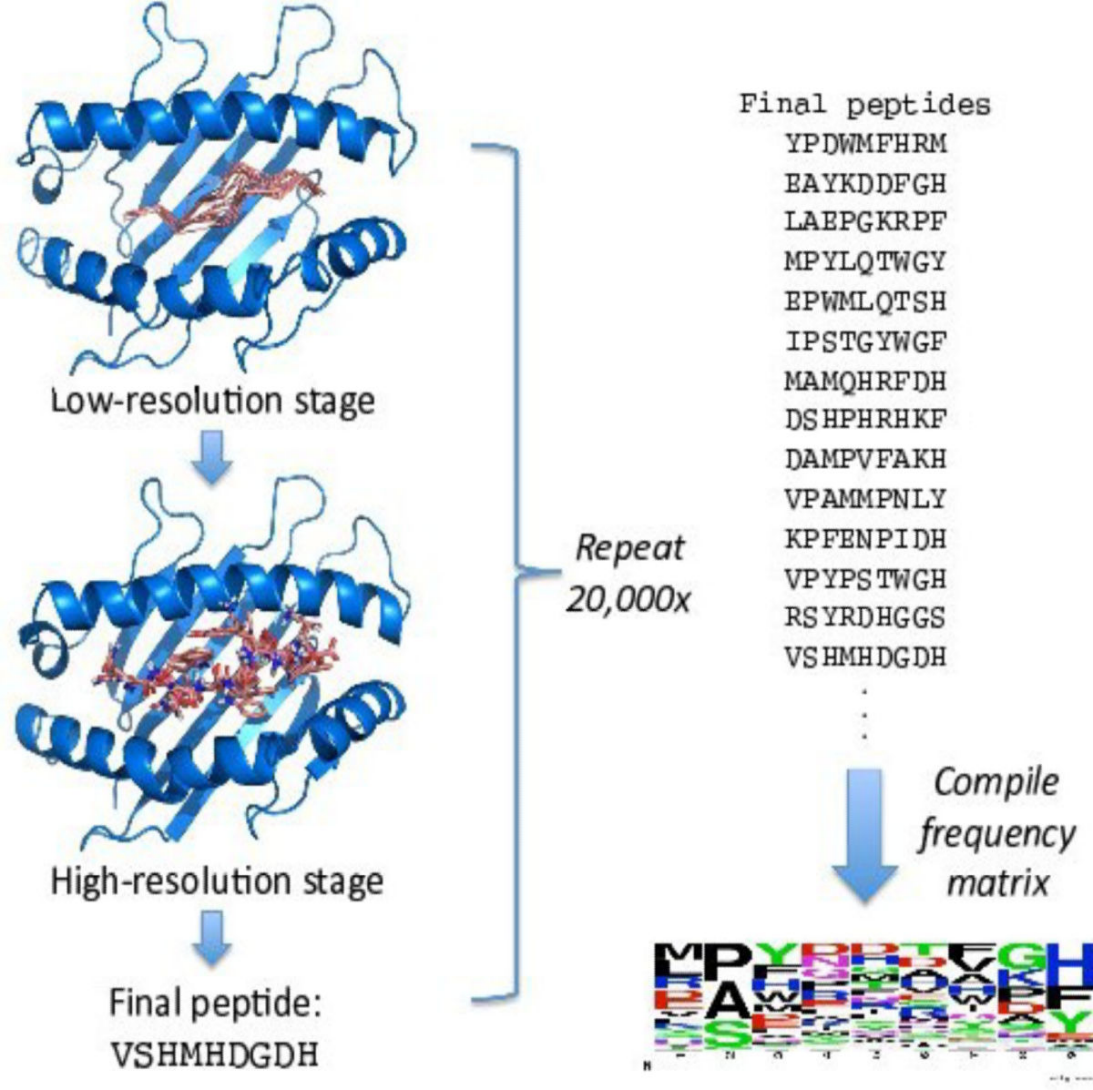
Structure-based prediction of peptide binding specificity. Two-stage modeling simulations explore peptide sequence and structure space to identify predicted high-affinity peptides. Binding specificity profiles are compiled from per-position amino acid frequencies in these sequences, and represented here as logo plots [19], in which the amino acid frequencies determine the height and order of the corresponding letters.

The low-resolution modeling stage is followed by a second, high-resolution refinement stage. In this stage, all sidechain atoms (including hydrogens) are added to the low-resolution backbone model, which is subsequently refined with a Monte Carlo plus Minimization (MCM) optimization procedure in a more realistic molecular mechanics force field [24]. Each move in this MCM optimization consists of a Monte Carlo perturbation to the current conformation or to the sequence of the peptide followed by gradient-based minimization; moves are accepted or rejected according to the Metropolis criterion [25]. The sequence of the peptide is sampled during this refinement simulation through the use of mutation MCM moves. The sequence of the peptide at the end of the refinement simulation is saved, and the list of all final peptide sequences is compiled into a per-position amino acid frequency profile, illustrated graphically by the logo representation [26] at the bottom right of Figure 6. Within each column the amino acids are ordered from top to bottom by frequency of occurrence; the height of each letter is proportional to the frequency of that amino acid in the PFM at that position.

## Comparing peptide binding profiles

**Figure 7:**
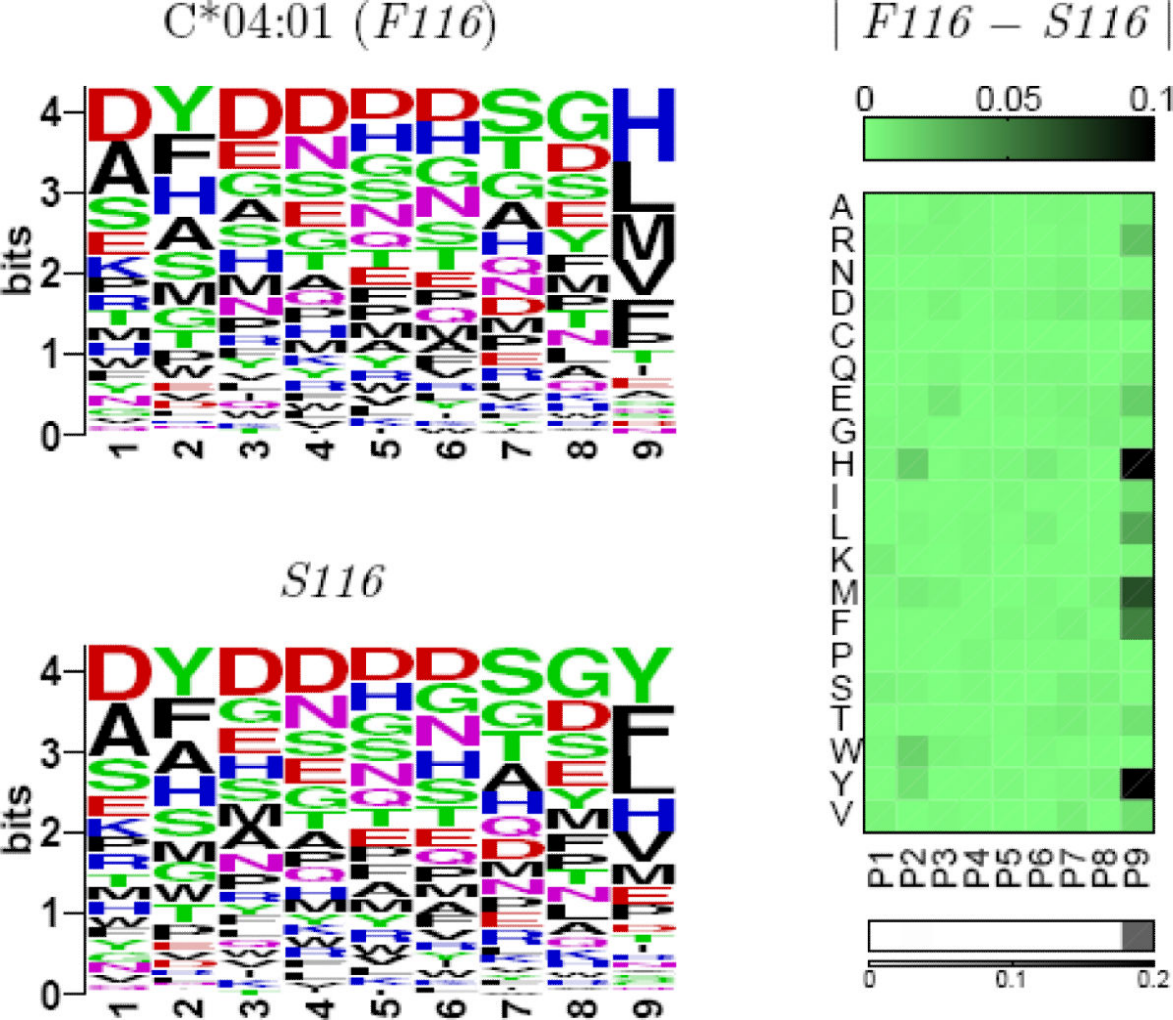
Prediction of binding specificity changes induced by the F116S amino acid mismatch. Predicted binding profiles are shown on the left as logo plots for the wild type C*04:01 template protein and for the same protein after mutating position 116 from F to S. Absolute changes in amino acid frequencies across all peptide positions are shown on the top right, colored from light green (no change) through black. The total change in the amino acid frequency distribution at each position, computed using the Jensen-Shannon divergence, is shown in the gray-scale bar on the bottom right.

Figure 7 illustrates the process of combining these modeling simulations to arrive at structure-based predictions of binding specificity divergence for amino acid mismatches. Starting from a “wild type” C*04:01 template with F at position 116, we conduct a binding specificity calculation as described above to arrive at the binding profile on the top left. We then mutate HLA position 116 to S, and recalculate a binding profile. This mutation slightly perturbs the wild type binding specificity profile by shifting the frequencies of certain amino acids at certain positions (for example, the frequency of Y is increased at position 9, corresponding to a predicted increase in affinity for Tyrosine at this position); the absolute magnitude of these frequency changes is shown on the top right of the figure. For each of the 9 positions in the profile, we compute the divergence between the wild type profile at that position and the mutant profile using the Jensen-Shannon divergence, a standard metric for comparing probability distributions [27]. This metric combines the differences in frequencies for each amino acid in the wild type and mutant profiles into a single number reflecting the overall divergence between the two amino acid frequency distributions. The results of these 9 comparisons are shown in the graphic on the bottom right, with white corresponding to little or no change in the binding preferences, and darker shades of gray reflecting larger changes. In this way we can see clearly that the predicted change in binding specificity induced by the F116S mutation is primarily localized to position 9 of the peptide. The sum of the 9 per-position divergences is taken as our prediction for the total binding specificity change induced by this mutation.

## Clinical dataset

We analyzed a dataset of 1,110 patient-donor pairs for which the patient and donor were mismatched at a single HLA-C allele, and matched at the other HLA-C allele and at the HLA-A, B, DRB1, and DQB1 loci (referred to as “9/10 pairs” for HLA-C); complete HLA-C protein sequences were available for each pair. Focusing on the 9/10 pairs facilitated identification of correlations between specific mismatches and outcome. As a measure of clinical outcome we focused on patient survival. Thus, as input for our analysis we had, for each patient-donor pair, two aligned HLA-C sequences and a single binary outcome variable. We mapped each pair to the set of residue-level mismatches (e.g., A/T at position 73, L/T at position 116, R/W at position 156, etc.) between their HLA-C proteins. We then tallied for each mismatch the total number of pairs with that mismatch, and the overall mortality rate for those pairs. We initially orientation in this analysis, i.e., we separated A/T mismatches at position 73 according to whether the patient had A and the donor T, or vice versa. This revealed striking asymmetry in mortality rates for a subset of the residue mismatches; for example, transplant pairs in which the patient had A and the donor had T at position 73 had a significantly lower mortality rate than pairs in which the patient had T and the donor had A. As our structure-based predictor of binding specificity divergence is inherently symmetric, we chose to symmetrize the mortality rates by taking the maximum of the two oriented mortality rates as the single mortality rate for each unoriented mismatch. In total, there were 90 distinct oriented amino acid mismatches and 50 unoriented mismatches observed in the clinical dataset.

Preliminary analysis revealed that certain alleles and allele pairs contributed disproportionately to the dataset, which had the effect of potentially skewing mortality rates for mismatches that were correlated with these alleles. For example, more than half of the 239 patient donor pairs with the 91 R/G mismatch had the C*03:03/C*03:04 allele-level mismatch. To reduce these biases, we computed revised mortality rates for each mismatch by randomly sub-sampling the patient-donor pairs contributing to that mismatch so that no single allele-pair contributed more than 10 pairs. This sub-sampling was repeated 1000 times and the calculated mortality rates were averaged to yield an adjusted risk score for each mismatch.

For assessing the impact of disease severity, we classified patients into three groups based on available clinical information. Diagnoses and stage of disease were categorized as low, intermediate, and high, with the high-risk category consisting of diseases that, on average, perform more poorly following transplant compared to the other two groups. The high-risk patients were taken to be any disease in relapse, CML in blast crisis, or MDS-RA with excess blasts or excess blasts in transformation.

## Amino acid mismatch set

**Figure 8:**
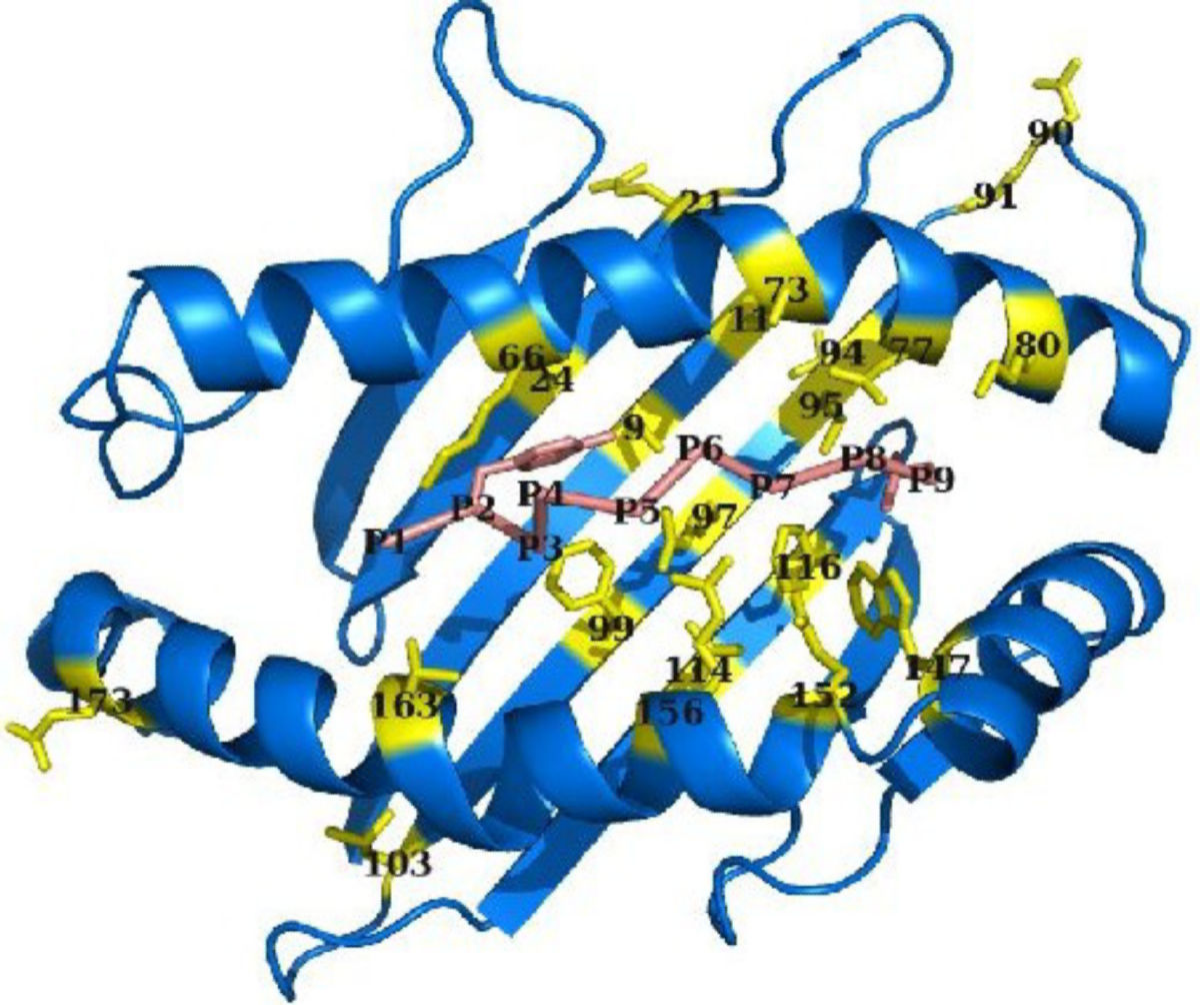
HLA mismatch positions analyzed in this study. The HLA molecule (residues 1-181 of PDB ID 1qqd, chain A) is colored blue, with mismatch positions colored yellow and shown in stick representation; the bound peptide (1qqd chain C) is shown in pink with the anchor residues in stick representation. All protein images generated with the molecular graphics package PyMol [28].

To reduce noise in the clinical risk factors, we restricted our analysis to high-counts mismatches, which we define as residue-level mismatches with at least 100 occurrences in the dataset in each orientation (patient/donor and donor/patient). We computed the predicted binding specificity divergence induced by a given mismatch by applying it within the context of a “wild-type” HLA protein of known three-dimensional structure, either C*03:04 or C*04:01 (PDB IDs 1efx [16] and 1qqd [17], respectively). For this study, we restricted ourselves to amino acid mismatches in which one of the two amino acids was found in the template protein, although this restriction (which eliminated only a single high-counts mismatch for each template) could easily be relaxed. For this reason, the specific set of mismatches analyzed for the two templates is slightly different. The complete set of mismatched positions is shown in Figure 8 (see also Supplementary Table S1). 

## Supplementary Material

Table of mismatches

## References

[1] Sasazuki T, Juji T, Morishima Y, Kinukawa N, Kashiwabara H, Inoko H, Yoshida T, Kimura A, Akaza T, Kamikawaji N, Kodera Y, Takaku F (1998). Effect of matching of class I HLA alleles on clinical outcome after transplantation of hematopoietic stem cells from an unrelated donor. Japan Marrow Donor Program.. N Engl J Med.

[2] Petersdorf EW, Anasetti C, Martin PJ, Gooley T, Radich J, Malkki M, Woolfrey A, Smith A, Mickelson E, Hansen JA (2004). Limits of HLA mis-match-ing in unrelated hematopoietic cell transplantation.. Blood.

[3] Flomenberg N, Baxter-Lowe LA, Confer D, Fernandez-Vina M, Filipovich A, Horowitz M, Hurley C, Kollman C, Anasetti C, Noreen H, Begovich A, Hildebrand W, Petersdorf E, Schmeckpeper B, Setter-holm M, Trachtenberg E, Williams T, Yunis E, Weisdorf D (2004). Impact of HLA class I and class II high-resolution matching on outcomes of unrelated donor bone marrow transplantation: HLA-C mismatching is associated with a strong adverse effect on transplantation outcome.. Blood.

[4] Lee SJ, Klein J, Haagenson M, Baxter-Lowe LA, Confer DL, Eapen M, Fernandez-Vina M, Flomenberg N, Horowitz M, Hurley CK, Noreen H, Oudshoorn M, Petersdorf E, Setterholm M, Spellman S, Weisdorf D, Williams TM, Anasetti C (2007). High-resolution donor-recipient HLA matching contributes to the success of unrelated donor marrow transplantation.. Blood.

[5] Ferrara GB, Bacigalupo A, Lamparelli T, Lanino E, Delfino L, Morabito A, Parodi AM, Pera C, Pozzi S, Sormani MP, Bruzzi P, Bordo D, Bolognesi M, Bandini G, Bontadini A, Barbanti M, Frumento G (2001). Bone marrow transplantation from unrelated donors: the impact of mismatches with substitutions at position 116 of the human leukocyte antigen class I heavy chain.. Blood.

[6] Kawase T, Morishima Y, Matsuo K, Kashiwase K, Inoko H, Saji H, Kato S, Juji T, Kodera Y, Sasazuki T (2007). High-risk HLA allele mismatch combinations responsible for severe acute graft-versus-host disease and implication for its molecular mechanism.. Blood.

[7] Hoof I, Peters B, Sidney J, Pedersen LE, Sette A, Lund O, Buus S, Niesen M (2009). NetMHCpan, a method for MHC class I binding prediction beyond humans.. Immunogenetics.

[8] Yanover C, Bradley P (2011). Large-scale characterization of peptide-MHC binding landscapes with structural simulations.. Proceedings of the National Academy of Sciences of the United States of America.

[9] Housset D, Malissen B (2003). What do TCR-pMHC crystal structures teach us about MHC restriction and alloreactivity?. Trends Immunol.

[10] Duquesnoy RJ (2002). HLAMatchmaker: a molecularly based algorithm for histocompatibility determination. I. Description of the algorithm.. Hum Immunol.

[11] Elsner HA, DeLuca D, Strub J, Blasczyk R (2004). HistoCheck: rating of HLA class I and II mismatches by an internetbased software tool.. Bone Marrow Transplant.

[12] Dudkiewicz M, Malanowski P, Czerwinacuteski J, Pawlstrokowski K (2009). An approach to predicting hematopoietic stem cell transplantation outcome using HLA-mismatch information mapped on protein structure data.. Biology of Blood and Marrow Transplantation.

[13] Duquesnoy R, Spellman S, Haagenson M, Wang T, Horowitz MM, Oudshoorn M (2008). HLAMatchmaker-defined triplet matching is not associated with better survival rates of patients with class I HLA allele mismatched hematopoietic cell transplants from unrelated donors.. Biol Blood Marrow Transplant.

[14] Duquesnoy RJ (2008). Clinical usefulness of HLAMatchmaker in HLA epitope matching for organ transplantation.. Curr Opin Immunol.

[15] Shaw BE, Barber LD, Madrigal JA, Cleaver S, Marsh SG (2004). Scoring for HLA matching? A clinical test of HistoCheck.. Bone Marrow Transplant.

[16] Boyington J, Motyka S, Schuck P, Brooks A, Sun P (2000). Crystal structure of an NK cell immunoglobulin-like receptor in complex with its class I MHC ligand.. Nature.

[17] Fan QR, Wiley DC (1999). Structure of Human Histocompatibility Leukocyte Antigen (Hla)-Cw4, a Ligand for the Kir2d Natural Killer Cell Inhibitory Receptor.. The Journal of Experimental Medicine.

[18] Nielsen M, Lundegaard C, Blicher T, Lamberth K, Harndahl M, Justesen S, Røder G, Peters B, Sette A, Lund O, Buus S (2007). NetMHCpan, a method for quantitative predictions of peptide binding to any HLA-A and -B locus protein of known sequence.. PLoS ONE.

[19] Crooks GE, Hon G, Chandonia JM, Brenner SE (2004). WebLogo: a sequence logo generator.. Genome Res.

[20] Gray JJ, Moughon S, Wang C, Schueler-Furman O, Kuhlman B, Rohl CA, Baker D (2003). Protein-protein docking with simultaneous optimization of rigid-body displacement and side-chain conformations.. J Mol Biol.

[21] Kuhlman B, Dantas G, Ireton GC, Varani G, Stoddard BL, Baker D (2003). Design of a novel globular protein fold with atomic-level accuracy.. Science.

[22] Simons KT, Kooperberg C, Huang E, Baker D (1997). Assembly of protein tertiary structures from fragments with similar local sequences using simlated annealing and Bayesian scoring functions.. J Mol Biol.

[23] Wang C, Bradley P, Baker D (2007). Protein-protein docking with backbone flexibility.. J Mol Biol.

[24] Misura KMS, Baker D (2005). Progress and challenges in high-resolution refinement of protein structure models.. Proteins.

[25] Metropolis NA, Rosenbluth AW, Rosenbluth NM, Teller AH, Teller E (1953). Equations of State Calculations by Fast Computing Machines.. J. Chem. Phys.

[26] Schneider TD, Stephens RM (1990). Sequence logos: a new way to display consensus sequences.. Nucleic Acids Res.

[27] Lin J (1991). Divergence measures based on the Shannon entropy.. IEEE Transactions on Information theory.

[28] Delano WL (2002). The PyMOL Molecular Graphics System.

